# Functional characterization of Arabidopsis phototropin 1 in the hypocotyl apex

**DOI:** 10.1111/tpj.13313

**Published:** 2016-10-14

**Authors:** Stuart Sullivan, Atsushi Takemiya, Eros Kharshiing, Catherine Cloix, Ken‐ichiro Shimazaki, John M. Christie

**Affiliations:** ^1^ Institute of Molecular, Cell and Systems BiologyCollege of Medical, Veterinary and Life SciencesUniversity of GlasgowBower BuildingGlasgowG12 8QQUK; ^2^ Department of BiologyFaculty of ScienceKyushu University744 Motooka, Nishi‐kuFukuoka819‐395Japan; ^3^ Department of BotanySt. Edmund's CollegeShillong793003MeghalayaIndia; ^4^ Present address: Graduate School of Sciences and Technology for InnovationYamaguchi University1677‐1 YoshidaYamaguchi753‐8512Japan; ^5^ Present address: Beatson Institute for Cancer ResearchGarscube Estate, Switchback RoadBearsden, GlasgowG61 1BDUK

**Keywords:** *Arabidopsis thaliana*, NPH3, phosphorylation, phototropin, phototropism, spatial expression

## Abstract

Phototropin (phot1) is a blue light‐activated plasma membrane‐associated kinase that acts as the principal photoreceptor for shoot phototropism in Arabidopsis in conjunction with the signalling component Non‐Phototropic Hypocotyl 3 (NPH3). *PHOT1* is uniformly expressed throughout the Arabidopsis hypocotyl, yet decapitation experiments have localized the site of light perception to the upper hypocotyl. This prompted us to investigate in more detail the functional role of the hypocotyl apex, and the regions surrounding it, in establishing phototropism. We used a non‐invasive approach where *PHOT1–GFP* (*P1–GFP*) expression was targeted to the hypocotyl apex of the phot‐deficient mutant using the promoters of *CUP‐SHAPED COTYLEDON 3* (*CUC3*) and *AINTEGUMENTA* (*ANT*). Expression of *CUC3::P1–GFP* was clearly visible at the hypocotyl apex, with weaker expression in the cotyledons, whereas *ANT::P1–GFP* was specifically targeted to the developing leaves. Both lines showed impaired curvature to 0.005 μmol m^−2^ sec^−1^ unilateral blue light, indicating that regions below the apical meristem are necessary for phototropism. Curvature was however apparent at higher fluence rates. Moreover, *CUC3::P1–GFP* partially or fully complemented petiole positioning, leaf flattening and chloroplast accumulation, but not stomatal opening. Yet, tissue analysis of NPH3 de‐phosphorylation showed that *CUC3::P1–GFP and ANT::P1–GFP* mis‐express very low levels of phot1 that likely account for this responsiveness. Our spatial targeting approach therefore excludes the hypocotyl apex as the site for light perception for phototropism and shows that phot1‐mediated NPH3 de‐phosphorylation is tissue autonomous and occurs more prominently in the basal hypocotyl.

## Significance Statement

Phototropism in Arabidopsis is mediated by the blue light receptor phototropin 1 (phot1), a plasma membrane‐associated kinase. Here we examined which tissues are important for establishing phototropism and other phot1‐mediated processes. Using targeted expression analysis of *PHOT1,* we excluded the hypocotyl apex as the site for light perception and showed that de‐phosphorylation of the phot1‐interacting protein NPH3 is tissue autonomous and occurs more prominently in the basal hypocotyl.

## Introduction

Light is an important environmental stimulus that regulates numerous aspects of plant growth and development. Phototropism, the re‐orientation of shoot growth towards a directional light source, is important during germination to promote light capture and early seedling growth, as well as photomorphogenesis (Christie and Murphy, 2013; Fankhauser and Christie, 2015). Traditionally, dark‐grown (etiolated) seedlings are used to study phototropism in both monocotyledonous and dicotyledonous species (Christie and Murphy, 2013). Recent studies using the model flowering plant, *Arabidopsis thaliana* have extended this analysis to light‐grown (de‐etiolated) seedlings which show retained phototropic responsiveness (Christie *et al*., 2011; Preuten *et al*., 2013, 2015) Yet, despite over a decade of research, the signalling mechanisms underlying this differential growth response remain largely unresolved.

Much of our understanding of the photodetection mechanisms responsible for phototropism has come from the isolation of Arabidopsis mutants with impaired phototropic responses (Sakai and Haga, 2012; Briggs, 2014; Liscum *et al*., 2014). Hypocotyl phototropism in Arabidopsis is induced by UV‐A/blue wavelengths (320–500 nm) and is perceived by plasma membrane‐associated photoreceptors known as the phototropins (Christie, 2007; Christie *et al*., 2015). Arabidopsis, like all flowering plants, contains two phototropins (phot1 and phot2) which overlap in function to regulate hypocotyl phototropism. Phot1 is the main phototropic receptor mediating curvature to low (≤1 μmol m^−2^ sec^−1^) and high (>1 μmol m^−2^ sec^−1^) fluence rates of blue light, whereas phot2 functions predominantly at higher light intensities (Sakai *et al*., 2001). Blue light detection via the cryptochrome blue light receptors also contributes to regulating phototropic responsiveness in Arabidopsis (Whippo and Hangarter, 2003). Similarly, it is well established that phytochrome can modulate hypocotyl curvature (Sakai and Haga, 2012; Goyal *et al*., 2013). For example, pre‐treatment of etiolated seedlings from above with red or blue light prior to directional blue light stimulation enhances the curvature response through the co‐action phyA (Sullivan *et al*., 2016a). Besides phototropism, phot1 and phot2 also act to control a range of other photomovement responses including leaf positioning, chloroplast relocation and stomatal opening (Christie *et al*., 2015), all of which contribute to optimising photosynthetic light capture and promote growth under low light conditions (Takemiya *et al*., 2005).

Phototropins are serine/threonine kinases that undergo autophosphorylation in response to blue light activation (Christie, 2007; Christie *et al*., 2015). The kinase domain of phototropin is located at the C‐terminus of the protein and belongs to the AGCVIII family (Willige and Chory, 2015). Although autophosphorylation occurs on multiple residues throughout the protein (Inoue *et al*., 2008a; Sullivan *et al*., 2008), phosphorylation of sites within the activation loop of the kinase domain have been reported to be essential for phototropin function (Inoue *et al*., 2008a, 2011). Light regulation of phototropin kinase activity is mediated by the N‐terminus of the protein which contains two specialised PAS domains designated LOV1 and LOV2 (Christie *et al*., 1999). Both light, oxygen, or voltage (LOV) domains serve as binding sites for the chromophore flavin mononucleotide, but several lines of evidence have shown that LOV2 functions as the predominant light sensor (Christie *et al*., 2002; Cho *et al*., 2007; Kaiserli *et al*., 2009). LOV1, conversely, appears to play a minor role in photodetection (Cho *et al*., 2007; Suetsugu *et al*., 2013), rather acting to modulate the regulatory action of LOV2 on phototropin kinase activity (Christie *et al*., 2002; Okajima *et al*., 2014).

While much is known with respect to phototropin activation by blue light, the signalling mechanisms following receptor autophosphorylation are less well defined. Phototropic curvature ultimately arises from an increase in cell elongation on the shaded side of the hypocotyl as a consequence of auxin accumulation (Christie and Murphy, 2013; Fankhauser and Christie, 2015). How phototropin coordinates this lateral redistribution of auxin in the hypocotyl remains unknown. Mutants lacking the phot1‐interacting protein, Non‐Phototropic Hypocotyl 3 (NPH3) are aphototropic (Liscum and Briggs, 1995) and fail to show lateral auxin accumulation in response to phototropic stimulation (Haga *et al*., 2005). Blue light activation of phot1 leads to rapid de‐phosphorylation of NPH3, which can be detected by immunoblotting owing to its enhanced electrophoretic mobility after blue light irradiation compared to a dark control (Pedmale and Liscum, 2007). Although the biological significance of this de‐phosphorylation is not known, NPH3 is proposed to regulate auxin redistribution through ubiquitin‐mediated proteolysis or re‐localization of target proteins involved in auxin transport (Roberts *et al*., 2011; Wan *et al*., 2012). A better understanding of NPH3 and its mechanism of action will therefore be key to unlocking the auxin transport mechanism(s) associated with phototropic growth and how these processes are initiated.

Imaging of auxin response sensors such as *DR5::GFP* has been used to assess the occurrence of auxin gradients in Arabidopsis hypocotyls following phototropic stimulation (Christie *et al*., 2011; Ding *et al*., 2011; Sakai and Haga, 2012). Initiation of lateral auxin gradients has been observed in the upper hypocotyl of de‐etiolated seedlings (Christie *et al*., 2011), implying that this region is important for light perception. Decapitation experiments concur with this conclusion as curvature is still observed when the cotyledons are excised (Christie *et al*., 2011), but is reduced when the cotyledonary node, including the shoot apical meristem (SAM) and leaf primordia are removed (Preuten *et al*., 2013). Similar decapitation experiments have been performed using etiolated seedlings and again localize the site of light perception to the upper hypocotyl (Preuten *et al*., 2013; Yamamoto *et al*., 2014).

Despite the importance of the upper hypocotyl in initiating phototropic growth, phot1 is localized throughout the seedling in Arabidopsis (Sakamoto and Briggs, 2002). However, expression of *PHOT1* within the upper hypocotyl, under the control of the *PHYTOCHROME KINASE SUBSTRATE 4 (PKS4)* promoter or the *CHLOROPHYLL A/B BINDING PROTEIN3* (*CAB3*) promoter, has been shown to be sufficient to restore a phototropic response in etiolated seedlings (Preuten *et al*., 2013). We therefore investigated how further restriction of *PHOT1* through tissues‐specific expression at regions within and surrounding the SAM, could impact its ability to initiate phototropic responses, as well as other phot1‐dependent processes in Arabidopsis.

## Results

### Expression and localization of *CUC3::PHOT1–GFP*

In order to target phot1 to the hypocotyl apex, the promoter of *CUP‐SHAPED COTYLEDON 3* (*CUC3*) was used to drive the expression of *PHOT1* translationally fused to the coding sequence of *GFP* (*CUC3::P1–GFP*). *CUC3* is a NAC (*NAM/ATAF1,2/CUC2*) transcription factor required for boundary and shoot meristem formation and is expressed in the seedling apex at the boundaries between the SAM and the cotyledons (Vroemen *et al*., 2003). The *CUC3::P1–GFP* construct was used to transform the *phot1 phot2* double mutant and three independent homozygous lines were isolated (1, 11 and 18).

**Figure 1:**
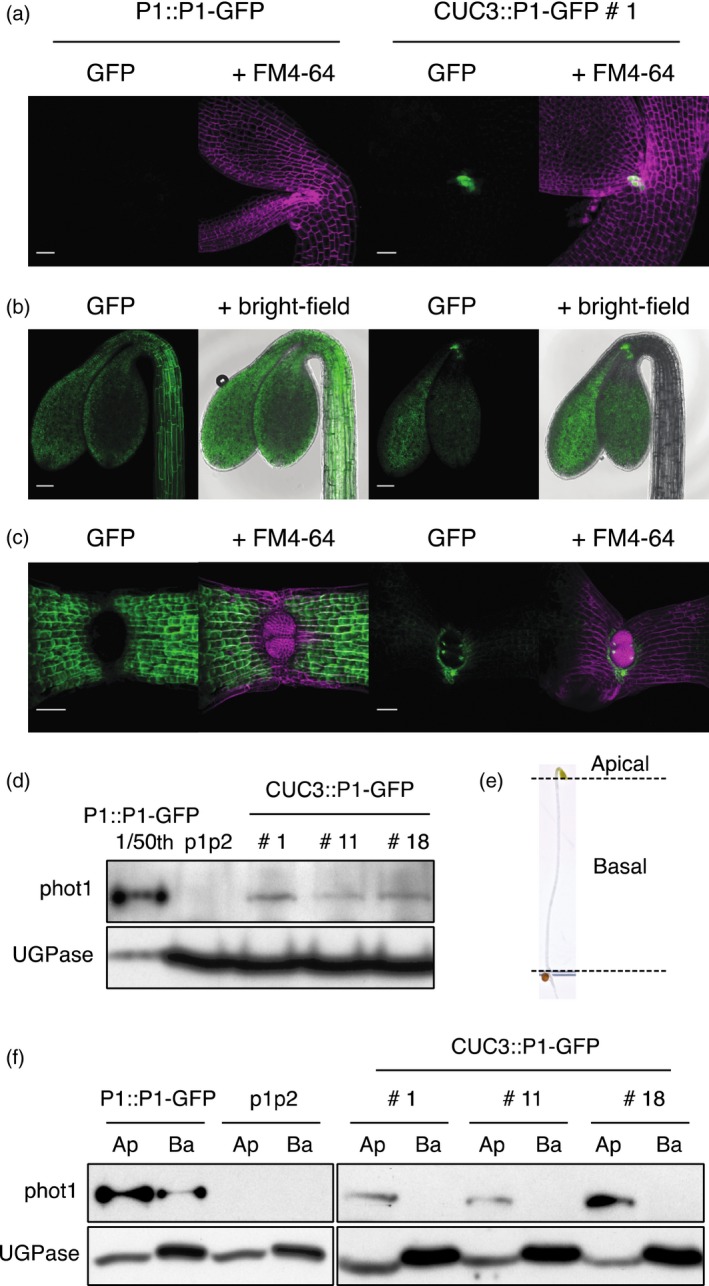
Expression and localization of *PHOT1::PHOT1‐* GFP (P1::P1–GFP) and *CUC3::PHOT1–GFP* (CUC3::P1–GFP) in transgenic lines. (a) Localization in embryos. SUM projection images of embryos expressing P1::P1–GFP or CUC3::P1–GFP. GFP is shown in green and FM4‐64 in magenta. Bar, 25 μm. (b) Localization in etiolated seedlings. SUM projection images of 3‐day‐old etiolated seedlings expressing P1::P1–GFP or CUC3::P1–GFP. GFP is shown in green and the bright‐field image in grey. Bar, 100 μm. (c) Localization in de‐etiolated seedlings. SUM projection images of 4‐day‐old de‐etiolated seedlings expressing P1::P1–GFP or CUC3::P1–GFP. GFP is shown in green and FM4‐64 in magenta. Bar, 50 μm. (d) Immunoblot analysis of total protein extracts from whole 3‐day‐old seedlings expressing P1::P1–GFP, three independent lines expressing CUC3::P1–GFP (lines 1, 11 and 18) and the *phot1 phot2* double mutant (p1p2). For P1::P1–GFP a 1 in 50 dilution of the total protein extract was loaded. Protein extracts were probed with anti‐phot1 antibody (phot1) and antibody raised against UDP‐glucose pyrophosphorylase (UGPase) as a loading control. (e) Picture of a 3‐day‐old etiolated seedling showing positioning of dissections of apical and basal segments. (f) Immunoblot analysis of total protein extracts from 3‐day‐old etiolated seedlings dissected into apical (Ap) and basal (Ba) segments. Protein extracts were probed with anti‐phot1 antibody (phot1) and antibody raised against UDP‐glucose pyrophosphorylase (UGPase) as a loading control.

Initially we compared the spatial localization of phot1–GFP in the *CUC3::P1–GFP* transgenic lines with *phot1 phot2* plants expressing phot1–GFP driven by the native *PHOT1* promoter (*P1::P1–GFP*) by confocal microscopy. Consistent with the known expression pattern of *CUC3* in embryos (Vroemen *et al*., 2003; Hibara *et al*., 2006), *CUC3::P1–GFP* was restricted to the embryonic apex at the junction with the developing cotyledons (Figures 1a and S1a). In contrast, no GFP signal could be detected in embryos expressing *P1::P1–GFP* indicating that phot1 is not expressed at this developmental stage. In etiolated seedlings, *CUC3::P1–GFP* was similarly expressed at the hypocotyl apex at the boundary of the SAM, with weaker expression also detectable within the cotyledons in each of the lines (Figures 1b and S1b). As previously reported, *P1::P1–GFP* is expressed throughout the hypocotyl and the cotyledons of etiolated seedlings (Sakamoto and Briggs, 2002; Wan *et al*., 2008). Due to the closed cotyledons of etiolated seedlings partially obscuring the GFP signal observed in the *CUC3::P1–GFP* seedlings, we also examined its localization in de‐etiolated seedlings with open cotyledons imaged from above (Figures 1c and S1c). Here, *CUC3::P1–GFP* expression can be seen as a ring of GFP signal surrounding the two developing leaves in de‐etiolated seedlings.

Immunoblot analysis of whole 3‐day‐old etiolated seedlings showed that phot1–GFP protein levels are significantly lower in all three *CUC3::P1–GFP* lines compared to *P1::P1–GFP* (Figure 1d), consistent with the restricted expression pattern observed by confocal microscopy. To further confirm that *CUC3::P1–GFP* expression was limited to the seedling apex, protein extracts were prepared from etiolated seedlings dissected into apical and basal sections. Apical segments comprised the upper hypocotyl including the cotyledons and apical hook, whereas basal segments consisted of the remainder of the hypocotyl above the shoot‐root transition zone (Figure 1e). Phot1–GFP was only detectable in protein extracts isolated from the apical segments in all three *CUC3::P1–GFP* transgenic lines, while phot1–GFP was apparent in both apical and basal segments in *P1::P1–GFP* seedlings (Figure 1f). In contrast to decapitation experiments (Christie *et al*., 2011; Preuten *et al*., 2013; Yamamoto *et al*., 2014), *CUC3::P1–GFP* seedlings offer a means to non‐invasively examine how localization in the hypocotyl apex, and to a lesser extent in the cotyledons, contributes to phot1 function.

### Assessment of phototropism in *CUC3::P1–GFP* etiolated seedlings

**Figure 2:**
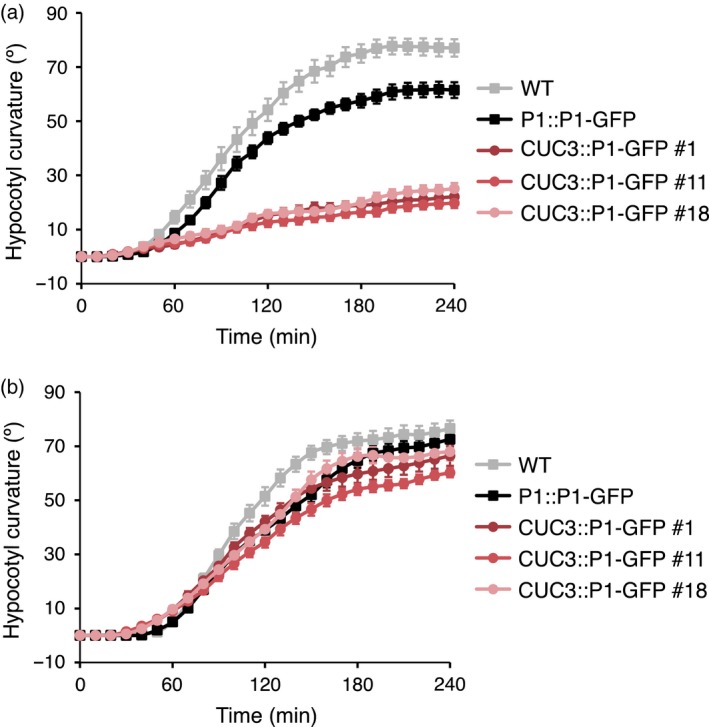
*CUC3::PHOT1–GFP* restores phototropism in the *phot1 phot2* double mutant under higher fluence rate blue light. Phototropism of 3‐day‐old etiolated wild‐type (WT) seedlings, seedlings expressing *PHOT1::PHOT1–GFP* (P1::P1–GFP) or three independent lines expressing *CUC3::PHOT1–GFP* (CUC3::P1–GFP lines 1, 11 and 18). (a) Seedlings irradiated with 0.005 μmol m^−2^ sec^−1^ of unilateral blue light for 4 h. (b) Seedlings irradiated with 0.5 μmol m^−2^ sec^−1^ of unilateral blue light for 4 h. Hypocotyl curvatures were measured every 10 min and each value is the mean ± standard error (SE) of 18–20 seedlings.

Having confirmed the localization of phot1–GFP to the hypocotyl apex in the *CUC3::P1–GFP* transgenic lines, we next assessed the ability of *CUC3::P1–GFP* to restore phototropism in the *phot1 phot2* double mutant background. Phot1 can mediate second‐positive phototropism even under very low fluence rates of blue light irradiation (Sakai *et al*., 2001). Therefore, continuous light‐induced second‐positive phototropism was examined by time‐lapse imaging of free‐standing etiolated seedlings irradiated with 0.005 μmol m^−2^ sec^−1^ of unilateral blue light for 4 h (Figure 2a). For wild‐type (WT) seedlings curvature commenced after ≈50 min of irradiation and reached an angle of ≈70° after 180 min. Phototropism was restored in seedlings expressing *P1::P1–GFP* with slightly delayed kinetics and reduced responsiveness compared to WT seedlings. These findings are however in agreement with previous publications showing that phot1–GFP exhibits somewhat reduced functionality for phototropism (Sakamoto and Briggs, 2002; Preuten *et al*., 2013). In contrast, *CUC3::P1–GFP* seedlings were greatly impaired in the magnitude of response under these light conditions indicating that restriction of phot1 to the hypocotyl apex, in addition to the cotyledons, is not sufficient to fully complement phototropism to very low fluence rates of unilateral blue light.

We also examined the phototropic responsiveness of *CUC3::P1–GFP* seedlings to higher fluence rates of unilateral blue light. Curvature in WT seedlings irradiated with low fluence rate blue light (0.5 μmol m^−2^ sec^−1^) commenced slightly later than when irradiated with 0.005 μmol m^−2^ sec^−1^ (Figure 2a,b), as has been reported recently (Haga *et al*., 2005). The reduced responsiveness of the *P1::P1–GFP* seedlings compared to WT seedlings was also apparent under low fluence rate blue light conditions, although pronounced curvatures were observed (Figure 2b). Although a minimal response was detected for *CUC3::P1–GFP* seedlings under very low blue light conditions, these were fully complemented for phototropism at higher light intensities, with kinetics similar to the *P1::P1–GFP* expressing seedlings. Thus, *CUC3::P1–GFP* is able to fully complement phototropism in the *phot1 phot2* double mutant but only under the higher blue light conditions examined.

### Localization of phot1‐mediated signalling in *CUC3::P1–GFP* seedlings

NPH3 is an essential component of the phototropic signalling pathway that couples blue light activation of the phototropins to the re‐orientation of hypocotyl growth. It is well established that NPH3 is rapidly de‐phosphorylated upon blue light irradiation in a phot1‐dependent manner (Pedmale and Liscum, 2007). Therefore, we investigated the phosphorylation status of NPH3 in response to blue light irradiation in dissected apical and basal segments of etiolated seedlings in order to gain a better understanding of how this phot1‐mediated signalling event is spatially initiated.

**Figure 3:**
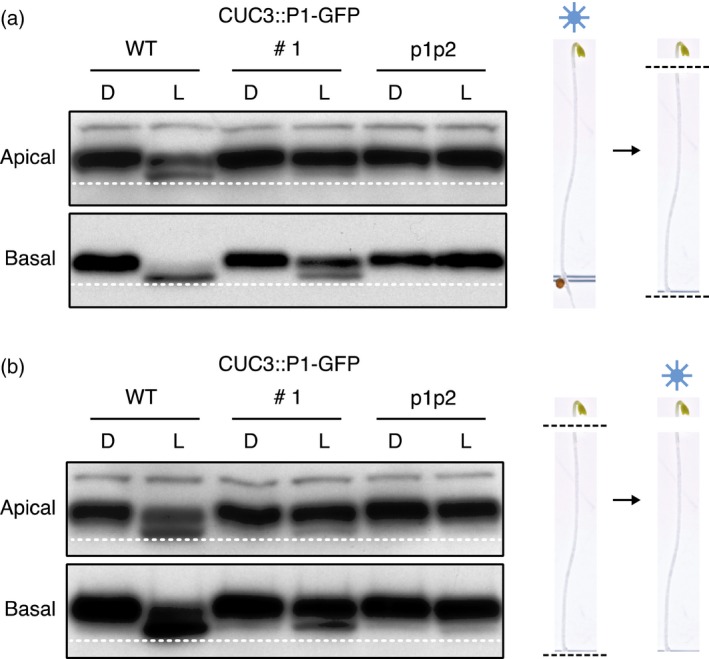
NPH3 phosphorylation status in apical and basal hypocotyl segments. Immunoblot analysis of total protein extracts from 3‐day‐old etiolated wild‐type (WT) seedlings, seedlings expressing *CUC3::PHOT1–GFP* (CUC3::P1–GFP line 1) or the *phot1 phot2* double mutant (p1p2). Seedlings were maintained in darkness (D) or irradiated with 20 μmol m^−2^ sec^−1^ of blue light for 15 min (L). (a) Seedlings were dissected into apical and basal segments after blue light irradiation. (b) Seedlings were dissected into apical and basal segments prior to blue light irradiation. Protein extracts were probed with anti‐NPH3 antibody. Dashed line indicates lowest mobility edge.

Etiolated seedlings either maintained in darkness (D) or irradiated with blue light (L; 20 μmol m^−2^ sec^−1^) were subsequently dissected into apical and basal segments under a dim red safe light. Immunoblot analysis of total protein extracts revealed an enhanced electrophoretic mobility of NPH3 in both apical and basal segments of light treated WT seedlings, but not in the *phot1 phot2* double mutant (Figure 3a), consistent with the localization pattern of phot1 in both these segments (Figure 1f). De‐phosphorylated NPH3 was more evident in the basal sections compared to the apical sections. Unexpectedly, in *CUC3::P1–GFP* seedlings (line 1) de‐phosphorylated NPH3 was barely detectable in the apical segments of blue light irradiated seedlings but was clearly visible in the basal segments (Figure 3a), a pattern that was opposite to the phot1 expression profile detected by GFP imaging (Figure 1a–c) and immunoblotting (Figure 1f).

Preuten *et al*., (2013) have proposed that the activation of phot1 in one cell layer is able to induce NPH3 de‐phosphorylation in all cell layers throughout the hypocotyl due to a mobile signal. In order to determine whether signalling events initiated in the hypocotyl apex of the *CUC3::P1–GFP* seedlings were able to induce NPH3 de‐phosphorylation in the basal segments of the hypocotyl, the above experiment was repeated except this time seedlings were dissected into apical and basal segments prior to irradiation (Figure 3b). Once again, immunoblot analysis showed that de‐phosphorylated NPH3 was clearly present in the basal segments of *CUC3::P1–GFP* seedlings (Figure 3b). Similar findings were also observed for the other *CUC3::P1–GFP* lines (Figure S2). This hypocotyl dissection analysis shows that the NPH3 de‐phosphorylation detected within the basal hypocotyl of *CUC3::P1–GFP* seedlings cannot be attributed to a mobile signal from the upper hypocotyl. Rather, a sufficient level of phot1–GFP must be present within the basal hypocotyl to mediate NPH3 de‐phosphorylation in this tissue, although this was not apparent from confocal microscopy or immunoblot analysis (Figure 1). Indeed, *PHOT1* transcripts were detectable in both apical and basal seedling segments of *CUC3::P1–GFP* seedlings by RT‐PCR analysis (Figure S3a).

### Complementation of phot1‐mediated responses in light‐grown plants

Phot1 mediates a variety of responses in plants which together promote plant growth through maximising light capture and optimising photosynthesis (Takemiya *et al*., 2005; de Carbonnel *et al*., 2010). These include petiole and leaf positioning and leaf expansion (Inoue *et al*., 2008b). Given the presence of phot1–GFP in the cotyledons of etiolated and de‐etiolated *CUC3::P1–GFP* seedlings (Figures 1b and S1b) and the detection of phot1 activity in the basal hypocotyl, as measured by NPH3 de‐phosphorylation (Figures 3 and S2), we examined the ability of *CUC3::P1–GFP* to complement these aforementioned responses.

**Figure 4:**
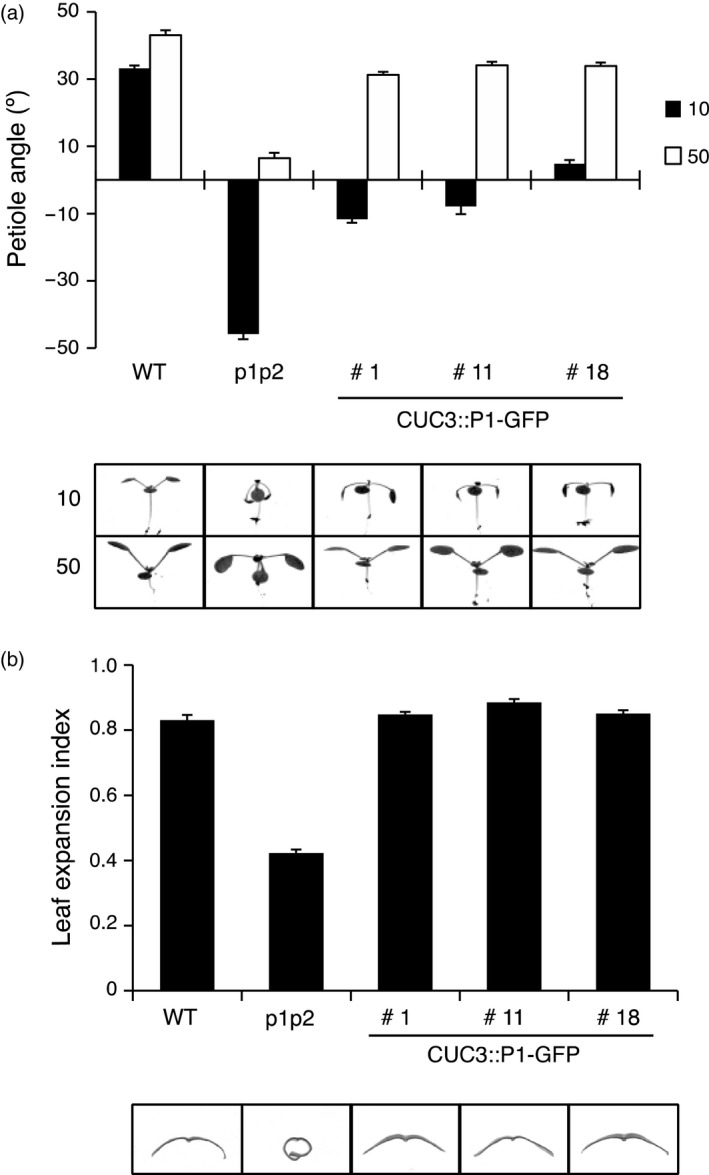
*CUC3::PHOT1–GFP* restores leaf positioning and leaf expansion in the *phot1 phot2* double mutant. Leaf positioning and leaf expansion of wild‐type (WT), *phot1 phot2* (p1p2) mutant and three independent lines expressing *CUC3::PHOT1–GFP* (CUC3::P1–GFP lines 1, 11 and 18). (a) Seedlings were grown on soil under white light at 80 μmol m^−2^ sec^−1^ for 7 days (16 h/8 h L/D cycle), then transferred to 10 μmol m^−2^ sec^−1^ (10) or 50 μmol m^−2^ sec^−1^ (50) white light (16 h/8 h L/D cycle) for 5 days before seedlings were photographed. Petiole angle from the horizontal was measured for the first true leaves. Each value is the mean ± standard error (SE) of 10 seedlings. (b) Plants were grown on soil under white light at 80 μmol m^−2^ sec^−1^ for 24 days (16 h/8 h L/D cycle.) The leaf expansion index of the 5^th^ rosette leaf was expressed as the ratio of the leaf area before and after artificial uncurling. Each value is the mean ± SE of 10 leaves. Images of leaf sections illustrate the leaf expansion phenotype.

We measured the petiole angle of the first true leaves of seedlings irradiated with low intensity (10 μmol m^−2^ sec^−1^) and moderate intensity (50 μmol m^−2^ sec^−1^) white light. In WT seedlings the petioles grew obliquely upwards under both light conditions, whereas in the petioles of *phot1 phot2* double mutant seedlings grew downwards under low white light and were horizontal under high white light (Figure 4a). *CUC3::P1–GFP* only partially restored leaf positioning in the *phot1 phot2* double mutant under low white light but fully restored leaf positioning under high white light irradiation (Figure 4a).

A characteristic feature of the *phot1 phot2* double mutant is the epinastic curled phenotype of the rosette leaves. In Arabidopsis, leaf expansion can be quantified by the leaf expansion index, which is the ratio of the leaf area measured before and after manual uncurling of the leaf (Takemiya *et al*., 2005). All three *CUC3::P1–GFP* lines fully complemented the *phot1 phot2* double mutant leaf expansion phenotype (Figure 4b). Consistent with this, *PHOT1* transcripts were readily detectable in rosette leaves of all three *CUC3::P1–GFP* lines (Figure S3b).

### *CUC3::P1–GFP* complements chloroplast accumulation but not stomatal opening

**Figure 5:**
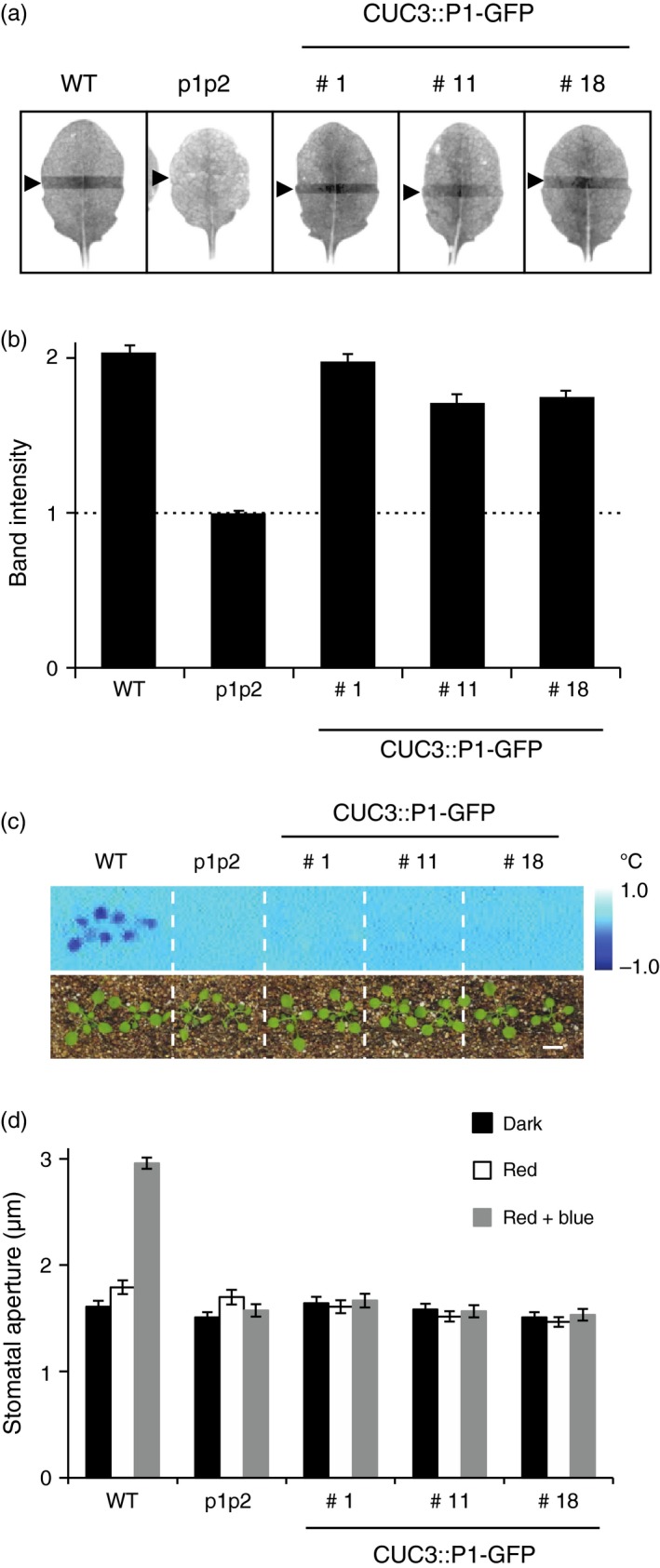
*CUC3::PHOT1–GFP* complements chloroplast accumulation but not stomatal opening in the *phot1 phot2* double mutant. (a) Slit band assays of chloroplast accumulation in wild‐type (WT), *phot1 phot2* (p1p2) mutant and three independent lines expressing *CUC3::PHOT1–GFP* (CUC3::P1–GFP lines 1, 11 and 18). Plants were grown on soil under white light at 80 μmol m^−2^ sec^−1^ for 3 weeks (16 h/8 h L/D cycle). Detached rosette leaves were placed on agar plates and irradiated with 1.5 μmol m^−2^ sec^−1^ blue light through a 1 mm slit for 1 h. Arrowheads indicate the irradiated areas. (b) Quantification of the slit band assays. The slit band intensity was quantified using ImageJ and the relative band intensities expressed as the ratio of the irradiated to the non‐irradiated areas. Ratios >1 indicate accumulation. The dashed line indicates a ratio of 1. Each value is the mean ± SE of 12 leaves. (c) Thermal images of wild‐type (WT), *phot1 phot2* (p1p2) mutant and three independent lines expressing *CUC3::PHOT1–GFP* (CUC3::P1–GFP lines 1, 11 and 18). Plants were irradiated with red light (80 μmol m^−2^ sec^−1^) for 50 min before 5 μmol m^−2^ sec^−1^ of blue light was superimposed. Images were obtained by subtracting an image taken under red light from one taken after 15 min of blue light irradiation. Lower panels show the plants. Bar, 1 cm. (d) Stomatal opening in of wild‐type (WT), *phot1 phot2* (p1p2) mutant and three independent lines expressing *CUC3::PHOT1–GFP* (CUC3::P1–GFP lines 1, 11 and 18). Epidermal strips from dark‐adapted plants were irradiated with red light (50 μmol m^−2^ sec^−1^) with or without blue light (10 μmol m^−2^ sec^−1^) for 2 h. Each value is the mean ± SE of 75 stomata, pooled from triplicate experiments.

Chloroplast accumulation is a cell‐autonomous response mediated by both phot1 and phot2 which allows plants to maximise light capture under low light conditions (Kong and Wada, 2014). The accumulation response can be visualised by the slit band assay, where a dark band appears on the leaf when irradiated with low fluence blue light (1.5 μmol m^−2^ sec^−1^) through a 1 mm slit (Suetsugu *et al*., 2005). A dark band was observed on the leaves of all three *CUC3::P1–GFP* expressing lines, similar to leaves from WT plants, whereas no response was observed in the *phot1 phot2* double mutant (Figure 5a,b). This ability to restore chloroplast accumulation movement further demonstrates expression of phot1–GFP in rosette leaves of *CUC3::P1–GFP* expressing plants (Figure S3b).

Phototropins also optimise photosynthesis by regulating stomatal opening in response to blue light (Kinoshita *et al*., 2001). Stomatal opening is accompanied by increased leaf transpiration, which results in a decrease in leaf temperature that can be monitored by infrared thermography (Takemiya *et al*., 2013). When WT plants were irradiated with 5 μmol m^−2^ sec^−1^ of blue light superimposed on a background of 80 μmol m^−2^ sec^−1^ red light, leaf temperature decreased by ≈1°C (Figure 5c). No change in leaf temperature was observed in *phot1 phot2* double mutant plants, or in the three lines expressing *CUC3::P1–GFP*. We also measured the stomatal aperture of epidermal strips in darkness, irradiated with red light or irradiated with red and blue light. Blue‐light‐induced stomatal opening was observed in epidermal strips from WT plants, but not in the *phot1 phot2* double mutant (Figure 5d). Furthermore, no change in stomatal aperture was observed in epidermal strips from plants expressing *CUC3::P1–GFP,* confirming the results obtained by infrared thermography.

### Localization and functionality of *ANT::P1–GFP*

In addition to targeting *PHOT1* expression to the hypocotyl apex, we also sought to localize phot1 further above the hypocotyl, to a region that would not be expected to restore phototropism in the *phot1 phot2* mutant. To achieve this, we chose the promoter of *AINTEGUMENTA* (*ANT*), an *APETALA2* (*AP2*)‐like transcription factor that is expressed in all organ primordia except roots (Elliott *et al*., 1996) and therefore would be expected to be only expressed in leaf primordia in young seedlings. The *ANT::P1–GFP* construct was introduced into the *phot1 phot2* double mutant and two independent homozygous lines were isolated (lines 2 and 4).

**Figure 6:**
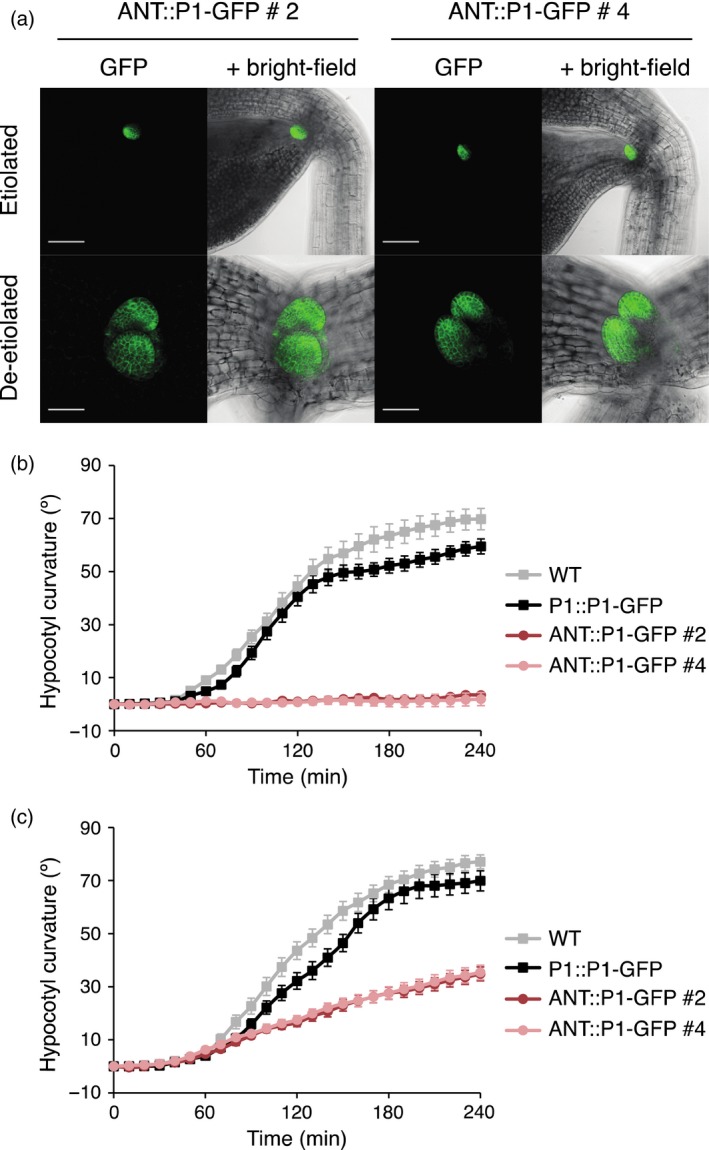
Localization and functionality of *ANT::PHOT1–GFP* (ANT::P1–GFP, lines 2 and 4) in transgenic lines. (a) Localization in etiolated and de‐etiolated seedlings. SUM projection images of 3‐day‐old etiolated seedlings (upper panels) and 4‐day‐old de‐etiolated seedlings (lower panels). GFP is shown in green and the bright‐field image in grey. Upper panels, bar 100 μm; lower panels, bar 50 μm. (b) Phototropism of 3‐day‐old etiolated wild‐type (WT) seedlings, seedlings expressing *PHOT1::PHOT1–GFP* (P1::P1–GFP) or two independent lines expressing *ANT::PHOT1–GFP* (ANT::P1–GFP lines 2 and 4). Seedlings irradiated with 0.005 μmol m^−2^ sec^−1^ of unilateral blue light for 4 h. Hypocotyl curvatures were measured every 10 min and each value is the mean ± standard error (SE) of 19–20 seedlings. (c) Phototropism of 3‐day‐old etiolated seedlings performed as in (b), except seedlings were irradiated with 0.5 μmol m^−2^ sec^−1^ of unilateral blue light.

Confocal microscopy with 3‐day‐old etiolated seedlings showed that localization of phot1–GFP was only observed in the developing leaves and this expression pattern was confirmed, and more clearly imaged in de‐etiolated seedlings viewed from above (Figure 6a). As somewhat expected from the results obtained from earlier decapitation experiments (Preuten *et al*., 2013; Yamamoto *et al*., 2014), both *ANT::P1–GFP* expressing lines were unable to mediate phototropic curvature under 0.005 μmol m^−2^ sec^−1^ of blue light irradiation (Figure 6b). However, both *ANT::P1–GFP* lines displayed measurable hypocotyl phototropism in response to continuous unilateral blue light irradiation at 0.5 μmol m^−2^ sec^−1^ (Figure 6c), although the magnitude and kinetics were greatly reduced compared to WT and *P1::P1–GFP* expressing seedlings.

We once again measured the phosphorylation status of NPH3 as a proxy for phot1 activity in *ANT::P1–GFP* seedlings to determine how this correlated with receptor localization. As with *CUC3::P1–GFP* seedlings (Figure 3b), hypocotyls from etiolated seedlings were dissected into apical and basal sections prior to blue light irradiation to stimulate NPH3 de‐phosphorylation. De‐phosphorylation of NPH3 was barely detectable in the apical segments, but was clearly visible in the basal hypocotyl of *ANT::P1–GFP* seedlings (Figure S4), although no phot1–GFP could be observed by confocal microscopy (Figure 6a). We therefore conclude that *ANT::P1–GFP* seedlings, similar to *CUC3::P1–GFP* seedlings, produce sufficient amounts of phot1–GFP within the basal hypocotyl to induce NPH3 de‐phosphorylation that is below the level of detection by confocal imaging.

## Discussion

Tissue‐specific localization of phytochrome and cryptochrome photoreceptors has proven to be a useful approach for identifying the site(s) of action of light‐mediated responses, as well as discriminating between local and long‐distance signalling pathways (Endo *et al*., 2005, 2007; Warnasooriya and Montgomery, 2009; Costigan *et al*., 2011; Kirchenbauer *et al*., 2016). Similar strategies have been applied to the study of phototropins. Kozuka *et al*., (2011) demonstrated that expression of *PHOT2* in mesophyll cells, but not in the epidermis, promoted palisade cell development in leaves in response to blue light. Likewise, spatial expression studies indicate that the action of phyA on phot1 signalling for phototropism occurs in tissues other than the epidermis (Kirchenbauer *et al*., 2016; Sullivan *et al*., 2016a). Preuten *et al*., (2013) recently showed that the expression of *PHOT1* in the upper hypocotyl under the control of the *PKS4* promoter was sufficient to restore phototropism in the *phot1 phot2* double mutant in response to 1.0 μmol m^−2^ sec^−1^ of unilateral blue light. *PHOT1* expression in the cotyledons and apical hook driven by the *CAB3* promoter also restored phototropism (Preuten *et al*., 2013). We therefore examined whether targeted expression of *PHOT1* to the hypocotyl apex using the *CUC3* promoter was sufficient to restore phototropism in the *phot1 phot2* double mutant. The *ANT* promoter was also used to express *PHOT1* in the developing leaves, above the SAM.

While phototropism was fully complemented in *CUC3::P1–GFP* seedlings irradiated with 0.5 μmol m^−2^ sec^−1^ of unilateral blue light, they showed only a marginal response at very low fluence rates (Figure 2), demonstrating that *CUC3::P1–GFP* is only partially functional for this response. Moreover, the detection of *PHOT1* transcripts and de‐phosphorylated NPH3 within the basal hypocotyl segments of *CUC3::P1–GFP* seedlings showed that *PHOT1* was more widely expressed in these seedlings than was evident by confocal microscopy or immunoblot analysis (Figures 1, S1 and S3a). *ANT::P1–GFP* was not expected to restore phototropism since *PHOT1* is not expressed in the hypocotyl (Figure 6a). Although *ANT::P1–GFP* seedlings were aphototropic under very low blue light (Figure 6b), they displayed a weak phototropic response at 0.5 μmol m^−2^ sec^−1^ (Figure 6c). However, NPH3 de‐phosphorylation was clearly visible in the basal hypocotyl segments of *ANT::P1–GFP* seedlings (Figure S4) implying that *PHOT1* was more widely expressed than was evident by confocal imaging (Figure 6a).

The results obtained in this study therefore highlight the difficulties and precautions that should be considered when ascribing the restoration of phototropism. In particular, mis‐expression of even very low levels of *PHOT1* could mediate phototropism depending upon the fluence rate of blue light used. Indeed, It has been reported previously that transgenic lines expressing *PHOT1* at levels significantly lower than wild‐type are fully complemented for phototropism (Christie *et al*., 2002; Doi *et al*., 2004; Cho *et al*., 2007; Preuten *et al*., 2013). However, the fluence rates used in these studies were 0.1 μmol m^−2^ sec^−1^ or higher. Based on our results, we propose that phototropism under very low fluence rates (such as 0.005 μmol m^−2^ sec^−1^) would provide a more discriminating test for functional complementation. Transgenic lines that mediate phototropism under these conditions could be viewed as fully‐complementing. In contrast, lines which only restore phototropism under higher fluence rates could arise from low levels of *PHOT1* expression. In the case here for *CUC3::P1–GFP* and *ANT::P1–GFP,* this is likely to arise from weak mis‐expression in other tissues/cell types. While *CUC3::P1–GFP* did promote a weak phototropic response under very low fluence rates (Figure 2a), our results obtained at very low fluence rates would indicate that expression of *PHOT1* at or above the SAM boundary was not sufficient to fully restore phototropism in the phot‐deficient mutant.

Our data also illustrates how NPH3 de‐phosphorylation can be used as a sensitive readout for detecting phot1 activity in different tissues. *CUC3::P1–GFP* and *ANT::P1–GFP* promoted less NPH3 de‐phosphorylation as compared to WT, which would be consistent with the very low phot1 levels in these lines. NPH3 de‐phosphorylation was also clearly evident in the basal hypocotyl segments from both *CUC3::P1–GFP* (Figure 3) and *ANT::P1–GFP* (Figure S4) seedlings even though phot1–GFP was not detected. At first, we rationalised that this basal NPH3 de‐phosphorylation could arise from long‐distance signalling from phot1 in the apical tissues. However, no difference in the degree of apical or basal NPH3 de‐phosphorylation was observed in *CUC3::P1–GFP* seedlings when dissections were performed either before or after blue light irradiation. These findings demonstrate the NPH3 de‐phosphorylation is tissue autonomous and argues against a mobile signal originating from phot1–GFP in hypocotyl apex.

We previously generated transgenic lines expressing *PHOT1–GFP* under the control of the epidermal‐specific promoter *MERISTEM LAYER 1* (*ML1*) in the *phot1 phot2* double mutant background (Sullivan *et al*., 2016a). Expression of *ML1::P1–GFP* was able to restore phototropism in response to 0.5 μmol m^−2^ sec^−1^ of unilateral blue light irradiation, however both the magnitude and kinetics of hypocotyl curvature were greatly reduced compared to *P1::P1–GFP* expressing seedlings (Sullivan *et al*., 2016a). NPH3, like phot1, is broadly expressed throughout the Arabidopsis hypocotyl (Preuten *et al*., 2013; Haga *et al*., 2015). We therefore examined *ML1::P1–GFP* seedlings for changes in NPH3 phosphorylation in response to blue light irradiation (Figure S5). Robust blue light‐induced NPH3 de‐phosphorylation was detected in total protein extracts isolated from *P1::P1–GFP* seedlings (Figure S5), whereas two bands corresponding to both the phosphorylated and de‐phosphorylated form of NPH3 were visible in two independent *ML1::P1–GFP* expressing lines (1M1 and 2A3). This pattern of NPH3 de‐phosphorylation would be expected if this process is restricted to the epidermis, rather than all cell layers. Taken together, these findings suggest that phot1‐mediated NPH3 de‐phosphorylation occurs locally in cells/tissues where both proteins are present. NPH3 de‐phosphorylation, combined with the high sensitivity of this response, also provides a useful means to assess the specificity of *PHOT1* expression when placed under the control of different promoters.

NPH3 de‐phosphorylation in WT seedlings occurs in both the apical and basal regions of etiolated seedlings (Figure 3) consistent with the expression of *PHOT1* in all tissues. De‐phosphorylation was found however to be more pronounced in the basal hypocotyl. A similar trend was observed in *CUC3::P1–GFP* and *ANT1::GFP* (Figures 3 and S4) seedlings. The phosphorylated form of NPH3 is proposed to be the active form in mediating hypocotyl phototropism (Haga *et al*., 2015). Hence, the lower level of NPH3 de‐phosphorylation in the apical region could reflect a requirement for active NPH3 in the upper hypocotyl to initiate phototropism. Higher levels of NPH3 de‐phosphorylation in the lower hypocotyl would also correlate with a lack of phototropic signalling in this region. Alternatively, the spatial difference in NPH3 de‐phosphorylation could arise from the optical properties of the tissues examined. A large proportion the apical segment examined consists of the cotyledons, which are far more opaque than the translucent hypocotyl which comprises the basal segment. Reduced light penetration of the cotyledons could therefore account for the lower levels of de‐phosphorylated NPH3 in the tissues. Further experiments will be required to differentiate between these possibilities.

Although the *CUC3* promoter was chosen to target *PHOT1* expression to the hypocotyl apex, phot1–GFP signals were also detected in the petioles and cotyledons via confocal microscopy (Figures 1b and S1b) and *PHOT1* transcripts were detected in rosette leaves (Figure S3b). Indeed, *CUC2* and *CUC3* have been shown to be expressed in the leaves where they are both involved in leaf serration (Nikovics *et al*., 2006; Hasson *et al*., 2011). This prompted us to assess the ability of *CUC3::P1–GFP* to restore other phot1‐mediated responses when expressed in the *phot1 phot2* double mutant background. Petiole positioning, leaf flattening and chloroplast accumulation to blue light intensities were all partially or fully complemented in the three independent *CUC3::P1–GFP* lines (Figures 4 and 5a,b), indicating the presence of phot1–GFP throughout the leaf tissue (Figure S3b). Petiole positioning, like phototropism, was only fully restored at higher fluence rates, possibly due to low phot1 protein levels in the cells/tissues required for this response. However, blue light induced stomatal opening was absent from the *CUC3::P1–GFP* lines (Figure 5c,d), indicating that the *CUC3* promoter does not lead to expression of phot1–GFP within the guard cells. In this regard, *CUC3::P1–GFP* lines phenocopy the *blue light signaling1* (*blus1)* mutant (Takemiya *et al*., 2013). *BLUS1* encodes a protein kinase that is directly phosphorylated by phot1, and whose activity is required for blue light induced stomatal opening. While *blus1* mutants are defective in phot1‐mediated stomatal opening, they are not impaired in phototropism, leaf flattening or chloroplast movements (Takemiya *et al*., 2013).

In conclusion, while our work emphasises important considerations when devising a promoter‐targeting approach to characterize phototropin function, the phototropism studies performed under very low fluence rates suggest that localization of phot1 to or above the hypocotyl apex is not sufficient to completely restore hypocotyl curvature in etiolated seedlings. A major challenge now will be to further define the region(s) within the upper hypocotyl where phot1 signalling is initiated and to decipher how NPH3 coordinates the changes in auxin accumulation that are ultimately required to promote this differential growth response.

## Experimental procedures

### Plant material and growth conditions

Wild‐type *Arabidopsis thaliana* (*gl‐1,* ecotype Columbia), the *phot1‐5 phot2‐1* double mutant and transgenic plants expressing *PHOT1::PHOT1–GFP/phot1‐5 phot2‐1* have been described previously (Kagawa *et al*., 2001; Sullivan *et al*., 2016a). Unless otherwise stated, seeds were planted on soil or surface sterilised and grown on vertically orientated plates containing half‐strength Murashige and Skoog (MS) medium with 0.8% agar (w/v). After cold treatment (4°C) for 2–4 days, seedlings were grown in a controlled environment room (Fitotron; Weiss‐Gallenkamp, Loughborough, UK) under 16 h 22°C: 8 h 18°C, light: dark cycles or maintained in darkness for etiolated seedlings. De‐etiolated seedlings were grown in darkness for 3 days and then transferred to 80 μmol m^−2^ sec^−1^ of white light in a 16 h: 8 h light: dark cycle for 1 day. Fluence rates for all light sources were measured with a Li‐250A and quantum sensor (LI‐COR, Cambridge, UK)

### Tissue‐specific expression of phototropin 1

The transformation vectors for *CUC3::PHOT1–GFP* and *ANT::PHOT1–GFP* were constructed using the modified binary expression vector pEZR(K)‐LN (Kaiserli *et al*., 2009). The *35S* promoter was removed using restriction sites *Sac*I and *Kpn*I and replaced with the promoter of *CUP‐SHAPED COTYLEDON 3* (*CUC3*) or *AINTEGUMENTA* (*ANT*), which were amplified from Columbia genomic DNA. The 5.1 kB *CUC3* promoter was amplified with primers pCUC3‐F (5′‐AAAAGAGCTCATCCTTACCTTTGCAAGAATTC‐3′) and pCUC3‐R (5′‐AAAAGGTACCCTTTTACTTAATATAACTGAAAAAG‐3′). The 5.1 kB *ANT* promoter was amplified with primers pANT‐F (5′‐AAAAGAGCTCCGTGACATATTGGCCTCGAT‐3′) and pANT‐R (5′‐AAAAGGTACCTTTGGTTTCTGCTTCTCTTCTTTCT‐3′). The full‐length coding sequence of *PHOT1* was amplified from cDNA and inserted using restriction sites *Kpn*I and *Bam*HI to generate *CUC3::PHOT1–GFP* or *ANT::PHOT1–GFP* constructs. The *phot1‐5 phot 2‐1* double mutant was transformed with *Agrobacterium tumefaciens* strain GV3101 as previously described (Davis *et al*., 2009). Based on the segregation of kanamycin resistance, independent T2 lines containing a single insertion were selected by confocal microscopy for tissue‐specific expression and resulting independent homozygous T3 lines were selected for analysis.

### Confocal microscopy

Localization of GFP‐tagged phot1 was visualised using a Zeiss LSM 510 or Leica SP8 laser scanning confocal microscope. For FM4‐64 staining, embryos dissected from developing seeds and apical segments of de‐etiolated seedling cut below the cotyledonary node were submerged in 8.2 μm FM4‐64 (Sigma‐Aldrich, Gillingham, UK) in distilled water for 10 min, rinsed in distilled water and observed immediately. The 488 nm excitation line was used; GFP fluorescence collected between 505–530 nm and FM4‐64 fluorescence collected between 560–615 nm. SUM projection images were constructed from z‐stacks using ImageJ software, version 2.0.0 (http://rsb.info.nih.gov/ij/).

### Immunoblot analysis

Total proteins were extracted from 3‐day‐old etiolated seedlings by directly grinding 100 seedlings in 100 μl of 2× SDS sample buffer. Dissection of seedlings into apical and basal segments was performed under a dissecting microscope with micro scissors (Fine Science Tools, Heidelberg, Germany) with red safe light illumination. Proteins were transferred onto polyvinylidene fluoride membrane (GE Healthcare, Little Chalfont, UK) by electroblotting and detected with anti‐phot1 polyclonal antibody (Cho *et al*., 2007), anti‐NPH3 polyclonal antibody (Tsuchida‐Mayama *et al*., 2008) and anti‐UGPase polyclonal antibody (Agrisera, Vännäs, Sweden). Blots were developed with horseradish peroxidase‐linked secondary antibodies (Promega, Southampton, UK) and Pierce ECL Plus Western Blotting Substrate (Thermo Fisher Scientific, Renfrew, UK).

### Phototropism

Phototropism of 3‐day‐old etiolated seedlings grown on a layer of silicon dioxide (Sigma‐Aldrich) watered with quarter‐strength MS medium was performed as previously described (Sullivan *et al*., 2016a). Images of seedlings were captured every 10 min for 4 h during unilateral illumination with 0.5 μmol m^−2^ sec^−1^ or 0.005 μmol m^−2^ sec^−1^ of blue light with a Retiga 6000 CCD camera (QImaging, Surrey, BC, Canada) connected to a PC running QCapture Pro 7 software (QImaging) with supplemental infrared light emitting diode (LED) illumination. Measurements of hypocotyl angles were made using ImageJ software.

### Petiole positioning and leaf expansion

For petiole positioning seedlings were grown on soil under 80 μmol m^−2^ sec^−1^ of white light in a 16 h: 8 h light: dark cycle for 7 days before being transferred to 10 m^−2^ sec^−1^ or 50 μmol m^−2^ sec^−1^ of white light for a further 5 days. One cotyledon was removed and seedlings were placed flat on agar plates and photographed. Petiole angles from the horizontal were measured using ImageJ software. Measurement of leaf expansion was carried out as described previously (Sullivan *et al*., 2016bb) from 4‐week‐old soil grown plants. Leaf areas were measured before and after uncurling and the ratio of the curled to uncurled area designated as the leaf expansion index. Leaf area was measured using ImageJ software.

### Chloroplast accumulation

Measurements of chloroplast accumulation were performed as described previously (Inoue *et al*., 2011; Sullivan *et al*., 2016bb). Band intensities were quantified using ImageJ software and the relative band intensities expressed as the ratio of the irradiated to the non‐irradiated areas.

### Thermal imaging and stomatal opening

Leaf temperature measurements by infrared thermography were preformed using a TVS‐8500 camera (NEC Avio Infrared Technologies, Tokyo, Japan) as described previously (Takemiya *et al*., 2013). Stomatal aperture measurements from the abaxial epidermis were performed as described previously (Takemiya *et al*., 2013) using an Eclipse TS100 microscope (Nikon, Tokyo, Japan).

### RNA extraction and RT‐PCR

Total RNA was isolated from 3‐day‐old etiolated seedlings dissected into apical and basal segments and rosette leaves from 3‐week‐old soil grown plants using the RNeasy Plant Mini Kit (Qiagen, Manchester, UK). Total RNA was DNase treated (Turbo DNA‐free; Thermo Fisher Scientific) and complementary DNA (cDNA) synthesised using random hexamers and SuperScript IV reverse transcriptase (Thermo Fisher Scientific). Reverse‐transcription PCR (RT‐PCR) was performed with GoTaq Hot Start Green Master Mix (Promega) and primers to amplify *PHOT1* and *ACTIN2* as described previously (Kaiserli *et al*., 2009).

## Author contributions

J.M.C., S.S. and K.I. designed and directed the research. S.S., A.T., E. K. and C.C. planned and performed experiments. All authors analysed the data. J.M.C. and S.S. wrote the manuscript. All authors commented on the manuscript.

## Supporting information


**Figure S1.** Localization of *CUC3::PHOT1–GFP* (CUC3::P1–GFP, lines 11 and 18) in transgenic lines.
**Figure S2.** NPH3 phosphorylation status in apical and basal hypocotyl segments.
**Figure S3.** RT‐PCR analysis of *PHOT1* transcripts.
**Figure S4.** NPH3 phosphorylation status in ANT::P1–GFP transgenic lines.
**Figure S5.** NPH3 phosphorylation status in ML1::P1–GFP transgenic lines.
