# Supplementary material for: Functional characterization of Arabidopsis phototropin 1 in the hypocotyl apex
Source: Plant J. 2016 Oct 14;88(6):907–20. doi: 10.1111/tpj.13313 (PMC5215551; doi:10.1111/tpj.13313)
Supplement: Supplementary file 1 — Figure S1. Localization of CUC3::PHOT1–GFP (CUC3::P1–GFP, lines 11 and 18) in transgenic lines. Figure S2. NPH3 phosphorylation status in apical and basal hypocotyl segments. Figure S3. RT‐PCR analysis of PHOT1 transcripts. Figure S4. NPH3 phosphorylation status in ANT::P1–GFP transgenic lines. Figure S5. NPH3 phosphorylation status in ML1::P1–GFP transgenic lines. [file TPJ-88-907-s001.docx]

**Supplementary Figure S1.** Localisation of *CUC3::PHOT1-GFP* (CUC3::P1-GFP, lines 11 and 18) in transgenic lines. **(a)** Localisation in embryos. SUM projection images of embryos expressing CUC3::P1-GFP. GFP is shown in green and FM4-64 in magenta. Bar, 25 μm. **(b)** Localisation in etiolated seedlings. SUM projection images of 3-day-old etiolated seedlings expressing CUC3::P1-GFP. GFP is shown in green and the bright-field image in grey. Bar, 100 μm. **(c)** Localisation in de-etiolated seedlings. SUM projection images of 4-day-old de-etiolated seedlings expressing *CUC3::P1-GFP*. GFP is shown in green and FM4-64 in magenta. Bar, 50 μm.

**Supplementary Figure S2.** NPH3 phosphorylation status in apical and basal hypocotyl segments. Immunoblot analysis of total protein extracts from 3-day-old etiolated seedlings expressing *CUC3::PHOT1-GFP* (CUC3::P1-GFP lines 11 and 18). Seedlings were maintained in darkness (D) or irradiated with 20 umol m^-2^ s^-1^ of blue light for 15 min. Seedlings were dissected into apical and basal segments after blue-light irradiation (L1) or prior to blue-light irradiation (L2). Protein extracts were probed with anti-NPH3 antibody. Dashed line indicates lowest mobility edge.

**Supplementary Figure S3.** RT-PCR analysis of *PHOT1* transcripts. *PHOT1* and control *ACTIN2* transcripts in wild-type (WT), the *phot1 phot2* double mutant (p1p2) and three independent lines expressing *CUC3::PHOT1-GFP* (CUC3::P1-GFP lines 1, 11 and 18). **(a)** Transcript levels in 3-day-old etiolated seedlings dissected into apical (Ap) and basal (Ba) segments. **(b)** Transcript levels in rosette leaves from plants grown on soil under white light at 80 μmol m^-2^ s^-1^ for 3 weeks (16/8 h L/D cycle)

**Supplementary Figure S4.** NPH3 phosphorylation status in ANT::P1-GFP transgenic lines. Immunoblot analysis of total protein extracts from 3-day-old etiolated wild-type (WT) seedlings and seedlings expressing *ANT::PHOT1-GFP* (ANT::P1-GFP lines 2 and 4). Seedlings were maintained in darkness (D) or irradiated with 20 umol m^-2^ s^-1^ of blue light for 15 min (L). Seedlings were dissected into apical and basal segments prior to blue-light irradiation. Protein extracts were probed with anti-NPH3 antibody. Dashed line indicates lowest mobility edge.

**Supplementary Figure S5.** NPH3 phosphorylation status in ML1::P1-GFP transgenic lines. Immunoblot analysis of total protein extracts from 3-day-old etiolated seedlings expressing *PHOT1::PHOT1-GFP* (P1::P1-GFP) or *ML1::PHOT1-GFP* (ML1::P1-GFP lines 1M1 and 2A3). Seedlings were maintained in darkness (D) or irradiated with 20 umol m^-2^ s^-1^ of blue light for 15 min. Protein extracts were probed with anti-NPH3 antibody. Dashed line indicates lowest mobility edge.
