# Supplementary material for: Functional characterization of Arabidopsis phototropin 1 in the hypocotyl apex
Source: Plant J. 2016 Oct 14;88(6):907–20. doi: 10.1111/tpj.13313 (PMC5215551; doi:10.1111/tpj.13313)
Supplement: Supplementary file 2 [file TPJ-88-907-s002.docx]

**Supporting Information Legends**

**Supplementary Figure S1.** Localisation of *CUC3::PHOT1-GFP* (CUC3::P1-GFP, lines 11 and 18) in transgenic lines. **(a)** Localisation in embryos. SUM projection images of embryos expressing CUC3::P1-GFP. GFP is shown in green and FM4-64 in magenta. Bar, 25 μm. **(b)** Localisation in etiolated seedlings. SUM projection images of 3-day-old etiolated seedlings expressing CUC3::P1-GFP. GFP is shown in green and the bright-field image in grey. Bar, 100 μm. **(c)** Localisation in de-etiolated seedlings. SUM projection images of 4-day-old de-etiolated seedlings expressing *CUC3::P1-GFP*. GFP is shown in green and FM4-64 in magenta. Bar, 50 μm.

**Supplementary Figure S2.** NPH3 phosphorylation status in apical and basal hypocotyl segments. Immunoblot analysis of total protein extracts from 3-day-old etiolated seedlings expressing *CUC3::PHOT1-GFP* (CUC3::P1-GFP lines 11 and 18). Seedlings were maintained in darkness (D) or irradiated with 20 umol m^-2^ s^-1^ of blue light for 15 min. Seedlings were dissected into apical and basal segments after blue-light irradiation (L1) or prior to blue-light irradiation (L2). Protein extracts were probed with anti-NPH3 antibody. Dashed line indicates lowest mobility edge.

**Supplementary Figure S3.** RT-PCR analysis of *PHOT1* transcripts. *PHOT1* and control *ACTIN2* transcripts in wild-type (WT), the *phot1 phot2* double mutant (p1p2) and three independent lines expressing *CUC3::PHOT1-GFP* (CUC3::P1-GFP lines 1, 11 and 18). **(a)** Transcript levels in 3-day-old etiolated seedlings dissected into apical (Ap) and basal (Ba) segments. **(b)** Transcript levels in rosette leaves from plants grown on soil under white light at 80 μmol m^-2^ s^-1^ for 3 weeks (16/8 h L/D cycle)

**Supplementary Figure S4.** NPH3 phosphorylation status in ANT::P1-GFP transgenic lines. Immunoblot analysis of total protein extracts from 3-day-old etiolated wild-type (WT) seedlings and seedlings expressing *ANT::PHOT1-GFP* (ANT::P1-GFP lines 2 and 4). Seedlings were maintained in darkness (D) or irradiated with 20 umol m^-2^ s^-1^ of blue light for 15 min (L). Seedlings were dissected into apical and basal segments prior to blue-light irradiation. Protein extracts were probed with anti-NPH3 antibody. Dashed line indicates lowest mobility edge.

**Supplementary Figure S5.** NPH3 phosphorylation status in ML1::P1-GFP transgenic lines. Immunoblot analysis of total protein extracts from 3-day-old etiolated seedlings expressing *PHOT1::PHOT1-GFP* (P1::P1-GFP) or *ML1::PHOT1-GFP* (ML1::P1-GFP lines 1M1 and 2A3). Seedlings were maintained in darkness (D) or irradiated with 20 umol m^-2^ s^-1^ of blue light for 15 min. Protein extracts were probed with anti-NPH3 antibody. Dashed line indicates lowest mobility edge.
